# White side test: A simple and rapid test for evaluation of nonspecific bacterial genital infections of repeat breeding cattle

**Published:** 2014

**Authors:** Fayaz Ahmad Bhat, Hiranya Kumar Bhattacharyya, Syed Akram Hussain

**Affiliations:** 1 *Department of Animal Reproduction, Gynecology and Obstetrics, Faculty of Veterinary Sciences and Animal Husbandry, Sher-e-Kashmir University of Agricultural Sciences and Technology of Kashmir, Shuhama-Alusteng, Srinagar, Jammu and Kashmir, India; *; 2 *Farm Science Centre, Assam Agricultural University, Dibrugarh, Assam, India; *; 3 *Department of Veterinary Public Health, Faculty of Veterinary Sciences and Animal Husbandry, Sher-e-Kashmir University of Agricultural Sciences and Technology of Kashmir, Shuhama-Alusteng, Srinagar, Jammu and Kashmir, India.*

**Keywords:** Bacterial culture, Cattle, Genital infection, Repeat breeding, White side test

## Abstract

The objective of the present study was to determine the grades of nonspecific bacterial infection of genitalia of repeat breeding cattle by a simple and rapid test under field condition. For this purpose, a total of 100 crossbred Jersey cows comprising of 80 repeat breeding animals presented for treatment and 20 normal cyclic (control group) animals presented for artificial insemination at their first service were selected. Estrual cervical mucus from all the animals was collected at 8 to 12 hr after the onset of behavioral estrus and subjected to white side test (WST) and bacteriological examination. The results of WST showed only 15% of control group had infection but the remaining 85% were free of it. In contrast, the majority of repeat breeding animals (57/80) showed infection (71.25%) and only 28.75% animals were free of infection. In bacterial culture, 60 (75.00%) from the 80 repeat breeding animals were found positive, and 20 (25.00%) were free of bacteria. All the three samples of control group that showed no color reaction in WST had also no growth in bacterial culture. The WST results showed a positive (*p* < 0.01) correlation of 0.48 with bacterial culture. It is thus concluded that under field condition WST can be used as a prime modality for ascertaining nonspecific bacterial infection of repeat breeding cattle before subjecting them to any antibiotic therapy thereby reducing the cost of diagnosis and treatment.

## Introduction

A repeat breeder is generally defined as a cow that has not conceived after three or more services exhibiting normal estrous cycle with apparently healthy genitalia.^1^ It is one of the important causes of infertility in cattle that results in delayed conception and increased calving interval, loss of milk production, reduction in calf crop, increased cost of treatment and culling of useful breeding animals leading to heavy economic losses to the dairy producers.^2^

The non-specific bacterial infection of the reproductive tract in cattle is the main cause of repeat breeding.^3^^-^^5^ The non-specific infectious agents during pre and postpartum periods frequently invade the uterus and produce metritis and endometritis leading to repeat breeding. Non-specific bacterial infections can cause inflammation of endo-metrium, denudation of its mucosa and change its secretion and thus alter uterine environment, resulting early embryonic death.^6^^,^^7^ Fertilization failure due to inflammatory exudates causing blockade of oviducts or death of sperms due to toxins produced by bacteria and inflammatory reaction before they reach site of fertilization also results from the non-specific bacterial infections. However, early embryonic death (< 42 days) is a major cause of conception failure in non-specific bacterial infections.^8^ The most commonly involved organisms are *Escherichia coli, Staphylococcus, Streptococcus, Corynebacterium, Bacillus, Pseudomonas, Micrococcus *and* Klebsiella*.^9^^,^^10^ The isolation and identification of the nonspecific organisms are not always possible because of high cost and it is also time consuming. On the other hand, frequent administration of new antibiotics and their indiscriminate use result in resistance of the organisms and their excessive use has a detrimental effect in the uterus. A very simple and very rapid test can alternatively be used to ascertain the grades of infection under field conditions and thereby restricting the unnecessary and indiscriminate use of antibiotics. The present study was therefore planned to evaluate the reliability of white side test (WST) in ascertaining genital infection of repeat breeding cattle reared in a rural tract of Kashmir valley. Novelty of this study was to correlate white side test with that of bacterial culture and thereby evaluating the reliability of this test in determining genital infection. The greatest advantage of this technique is to ascertain grades of infection within only few minutes. 

## Materials and Methods

The present study was conducted at Teaching Veterinary Clinical Complex, Faculty of Veterinary Sciences and Animal Husbandry, Sher-e-Kashmir University of Agricultural Sciences and Technology of Kashmir, Srinagar, India, during the period from October 2009 to December 2010. A number of 100 crossbred Jersey cattle comprising of 80 repeat breeding cows presented for treatment from nearby villages and 20 clinically normal cows presented for artificial insemination at their first service, were selected. Cows that failed to conceive in three or more regular services with apparently healthy genitalia were considered as repeat breeders.^1^ Detailed gynecoclinical examination was carried out to all the repeat breeding animals and those with gross genital pathology were excluded from the study^11^ and were replaced with other repeat breeding animals. Clinically healthy animals presented for insemination within 60 to 90 days after normal parturition constituted the control group. 

Estrual cervical mucus from all the animals was collected at 8 to 12 hr after the onset of behavioral estrus. Estrual mucus was collected as described by Dhillon *et al*. with little modification to make it more field applicable.^12^ For this purpose; animals were properly restrained in the service crate. Lubricated (with sterile glycerine) full sleeve gloved left hand was inserted per rectum and back racking was done for evacuation of rectal feces. Perineal region including vulva was thoroughly washed with soap and running tap water, dried with soft absorbable cotton, disinfected with 70% alcohol. After 15 to 20 min, disinfection of area was repeated with 70% alcohol while both the vulvar lips were held apart by an assistant. A sterilized 10 mL glass pipette with pointed end directed outwards was inserted in the vagina. The external pointed end of the pipette was connected to a 20 mL disposable syringe with a rubber adopter for aspiration. The pipette was guided through the vaginal canal to the cervical os or mid cervix by the left hand already introduced per rectum. Cervical mucus was aspirated from the os or mid cervix and then transferred to a sterilized test tube.

For conducting WST, 1 mL of estrual cervical mucus was heated with equal volume of 5 to 10% sodium hydroxide (Merck, Mumbai, India) up to boiling point and after cooling the intensity of color changes were studied and graded as normal (turbid or no color), mild infection (light yellow color), moderate infection (yellow color) and severe infection (dark yellow color).

The isolation and identification of the organisms were carried out on the basis of cultural, morphological, colony characteristics, motility and biochemical reactions.^13^

The data recorded for different parameters were shown in percentage. The correlation between bacterial culture and results of WST was calculated by Spearman’s rank correlation.

## Results

The results of WST revealed no infection in 28.75% animals of repeat breeding and 85% animals of control group (Table 1). The intensity of color changes showing grades of infection are shown in Table 1. 

**Table 1:** Results of white side test showing grades of infection based on color intensity between repeat breeders and normal cyclic (Control group) animals.

**Colour Intensity**	**Grades of Infection**	**Repeat breeding**		**Control group**	
		**n**	**(%)**	**n**	**(%)**
No color	0 (Normal)	23	28.75	17	85
Light yellow	+ (Mild)	46	57.50	3	15
Yellow	++ (Moderate)	6	7.50	0	0
Dark yellow	+++ (Severe)	5	6.25	0	0
Overall		80 (100)		20 (100)	

In bacterial culture, 60 (75.00 %) from the 80 repeat breeding animals were found positive and 20 (25.00 %) were free of bacteria (Table 2). The different bacterial isolates from repeat breeding cows were *Staphylococcus spp*. 16 (21.05%), *E. coli* 14 (18.42%), *Bacillus *spp. 10 (13.16%), *Corynebacterium *spp. 10 (13.16%), *Pseudomonas *spp. 8 (10.53%), *Proteus *spp. 8 (10.53%), *Klebsiella *spp. 6 (7.89%), and *Streptococcus *spp. 4 (5.26%). 

On the other hand, out of 20 samples collected from the animals of control group only 3 (25.00%) showed bacterial growth and remaining 17 (85.00%) were free of bacterial isolates. All the three positive samples revealed only single isolate, (Table 2).

Results of WST showed a positive correlation of 0.48 with bacterial culture (*p* < 0.01) and thereby showing reliability of this simple test under field condition.

**Table 2:** Results of bacterial culture between repeat breeding and normal cyclic (Control group) animals

**Parameters**	**Repeat breeding**			**Control group**	
	**n**	**(%)**		**n**	**(%)**
Number of samples positive for bacterial isolates	60	75.00		3	15.00
Number of samples free from bacterial isolates	20	25.00		17	85.00
Number of samples having single bacterial isolate	46	76.67		3	100.0
Number of samples having two bacterial isolates	12	20.00		0	0.00
Number of samples having three bacterial isolates	2	3.33		0	0.00
Overall number of samples having mixed infection	14		23.33	0	0.00

## Discussion

From the results of WST it has been inferred that only 15.00% animals of control group showed infection and remaining 85.00% animals were free of infection; however majority of repeat breeding animals showed infection (71.25%) and only 28.75% animals were free of infection. Satheshkumar and Punniamurthy reported color reaction on WST in 92.85% cervical mucus samples of normal animals indicating subclinical uterine infections.^14^ Normal, mild, moderate and severe color changes in WST of cervical mucus of 26 repeat breeding cows were 0 (0.00%), 12 (46.15%), 10 (38.46%) and 4 (15.38%), respectively.^15^ Methai *et al*. recorded color changes in 100% samples of cervical mucus in repeat breeding cows suffering from endometritis.^16^

Bacteriological examination revealed that 75.00% (60/ 80) samples yielded bacterial isolates while as 25.00% (20/80) samples were free of bacteria. The results of the present study were more or less in agreement with the findings of previous workers.^17^^,^^18^ Bacterial infection of 80 to 100% in the cervical mucus of repeat breeding animals has also been reported.^9^^,^^19^^,^^20^ Gani* et al*. recorded only 62.2% bacterial infection in cervical mucus of repeat breeding animals.^10^ Difference in the rate of infection recorded in different studies might be due diversity in the severity of infection and variations in agro-climatic conditions of the areas where those studies were undertaken. 

Single organism was isolated from 46 (76.67%), whereas mixed infections consisting of more than one type of organisms were observed in 14 (23.33%) of repeat breeding cows in the current study. This finding was more or less in accordance with the findings of Chandrakar *et al*. and Mane *et al*. who reported 70 and 80.00% samples with single isolate and 30 and 20.00% samples with multiple isolates.^19^^,^^20^ Several other authors also reported single isolate cases dominated multiple isolates in samples obtained from repeat breeding animals.^9^^,^^18^^,^^20^ However, Javed and Khan recorded single isolate in 45.45 % and mixed isolates in remaining samples.^21^

Out of 20 samples from the animals of control group only 3 (15.00 %) showed bacterial growth on the culture, whereas 17 (85.00%) samples did not show any bacterial growth similar to the results of WST. This finding corroborates earlier findings that normal animals may also contain infections.^22^^,^^23^ Some earlier workers could obtain complete sterile cervical mucus from normal breeding cows.^19^^,^^20^ All the positive samples in this study revealed single isolate. Almost similar findings in normal breeding cows were reported by Das *et al*.^24^

Results of WST showed a positive correlation of 0.48 with bacterial culture indicating reliability of WST with that of bacterial culture (*p* < 0.01). This finding underlies the importance of WST under field conditions where optimum facility for microbial isolation is not available. The WST can also be used as a compulsory test to determine grades of nonspecific genital infections in repeat breeding cattle before subjecting them to any antibiotic therapy.
